# Multielectrode Radiofrequency Ablation for Resectable Metachronous Liver Metastasis from Colorectal Cancer

**DOI:** 10.3390/jcm10163712

**Published:** 2021-08-20

**Authors:** Hou-Ying Cheng, Kai-Wen Huang, Jin-Tung Liang, Been-Ren Lin, John Huang, Ji-Shiang Hung, Chi-Ling Chen

**Affiliations:** 1 Division of General Surgery, Department of Surgery, National Taiwan University Hospital, Taipei 100229, Taiwan; 107210@ntuh.gov.tw; 2 Graduate Institute of Clinical Medicine, College of Medicine, National Taiwan University, Taipei 100229, Taiwan; chlnchen@ntu.edu.tw; 3 Division of Colorectal Surgery, Department of Surgery, National Taiwan University Hospital, Taipei 100229, Taiwan; jintung@ntu.edu.tw (J.-T.L.); beenrenlin@ntu.edu.tw (B.-R.L.); docjohn@ntuh.gov.tw (J.H.); jshung@ntu.edu.tw (J.-S.H.)

**Keywords:** colorectal cancer, hepatectomy, liver metastasis, propensity-score matching, radiofrequency ablation, switching controller

## Abstract

The outcome of radiofrequency ablation (RFA) for liver metastases from colorectal cancer (CRLM) has been thought to be inferior to metastasectomy. However, the recent development of multielectrode RFA (multi-RFA) systems has made the ablation zone larger and more complete. Thus, we assessed the survival benefits of this modality in cases of metachronous CRLM. This retrospective study assessed patients diagnosed with resectable metachronous CRLM between 2013 and 2016; 132 patients were categorized by treatment for liver metastases: multi-RFA (*n* = 68), hepatectomy (*n* = 34), or systemic treatment only (*n* = 30). Therapeutic effectiveness, outcomes, and intervention-related complications were compared between groups. Median overall survival (OS), recurrence-free survival (RFS), and intrahepatic recurrence-free survival (IHRFS) were 69.8, 85.2, and 59.7 months for the hepatectomy group; 53.4, 41.3, and 32.3 months for the multi-RFA group; and 19.1, 7.1, and 7.1 months for the systemic treatment group. No significant differences were observed between the multi-RFA and hepatectomy groups after a median follow-up of 59.8 months. This study demonstrated that multi-RFA and hepatectomy provide similar survival benefits for patients with resectable CRLM. Multi-RFA may represent a reliable treatment option for the management of resectable liver metastases.

## 1. Introduction

Colon cancer is the third most frequently diagnosed cancer and has the second highest mortality rate worldwide, accounting for one-tenth of all cancer cases and deaths annually [1]. Colon cancer is the most common form of cancer in Taiwan [2], and the liver is the most common metastatic site [3]. Approximately 25% of patients with colon cancer have liver metastases (CRLM) at initial diagnosis, and 50% develop metachronous liver metastases [4]. Liver metastasis can develop synchronously or metachronously. The relatively poor prognosis of synchronous metastatic liver disease has been suggested to be attributed to the fact it is more disseminated than metachronous metastatic liver disease [5].

The median survival of patients with CRLM receiving palliative care is between 7 and 8 months [6]. The 5-year overall survival rate following surgical resection for CRLM is 24–40%, with a median survival of 28–46 months [7]. The rate of recurrence is high (48–80%), and 75% of recurrences occur within two years of hepatectomy [8]. Nonetheless, surgery remains the gold standard treatment for resectable CRLM [9].

Radiofrequency ablation (RFA) destroys cancer tissues by generating heat through application of an alternating high-frequency electric current (450–500 kHz) [10] and has been employed for the management of small hepatocellular carcinoma for decades. Recently, RFA has also been increasingly used as an alternative to surgery for CRLM and has been shown to improve survival in several studies [11]. The current NCCN guidelines already recommend that RFA should be considered in cases with CRLM, whereas resection is still the preferred option for metachronous CRLM [12]. 

The long-term effects of hepatic resection are well established; however, the efficacy, utility, and outcomes of RFA have yet to be fully elucidated in high-quality clinical trials [13,14]. Previous studies have suggested that RFA is inferior to hepatic resection in terms of survival and recurrence outcomes, despite a lower risk of complications and better postprocedural quality of life [15]. 

RFA with switching-controlled multielectrodes, internal cooling systems, and sophisticated imaging guidance has recently made it possible to create a larger yet better-defined ablation zone with less pronounced heat-sink effects imposed by adjacent vessels. Advanced multielectrode RFA (multi-RFA) is well suited to the resection of large liver metastases without increasing the risks of incomplete ablation and local recurrence [16].

In this study, we aimed to evaluate the efficacy of multi-RFA with a multielectrode system as an integrative treatment modality for resectable metachronous liver metastases in patients with colorectal cancer. The secondary objective was to identify the characteristics of patients with CRLM who are most likely to benefit from multi-RFA treatment. 

## 2. Patients and Materials

**Figure 1:**
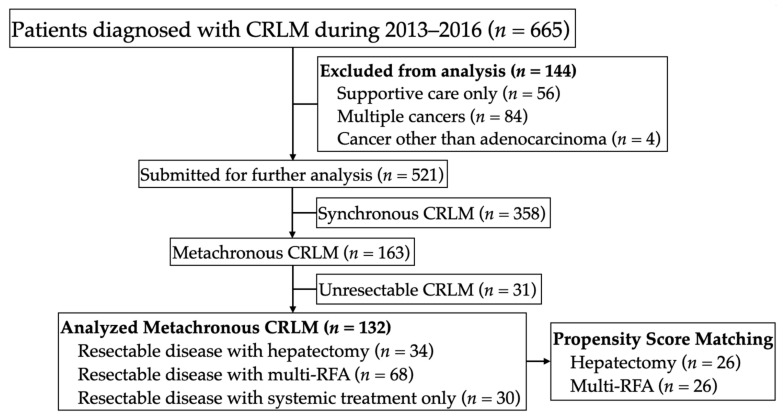
CONSORT flow diagram of the study participants.

This study was approved by the Ethics Committee of the National Taiwan University Hospital in accordance with all relevant guidelines. Data from patients diagnosed with resectable metachronous CRLM at our institute were retrospectively collected for the period between January 2013 and December 2016. We identified patients with resectable metachronous liver metastases due to colorectal cancer and grouped these patients according to the treatment used for liver metastases: hepatectomy, multi-RFA, or systemic treatment only (Figure 1). Patients with extrahepatic disease were not excluded from the study. In each case, the liver metastases were evaluated in terms of resectability by retrospectively reviewing preoperative medical images in order to confirm the feasibility of complete macroscopic resection while preserving at least 30% of the liver [17,18].

All of the patients in this study had colorectal tumor resection and received empirical systemic therapy in accordance with NCCN guidelines. The therapeutic options provided by physicians for liver metastasis included radical hepatectomy and multi-RFA with intention to curative treatment as an alternative modality, and the decision was made based on the discussion of the patient, family, and physicians.

**Figure 2:**
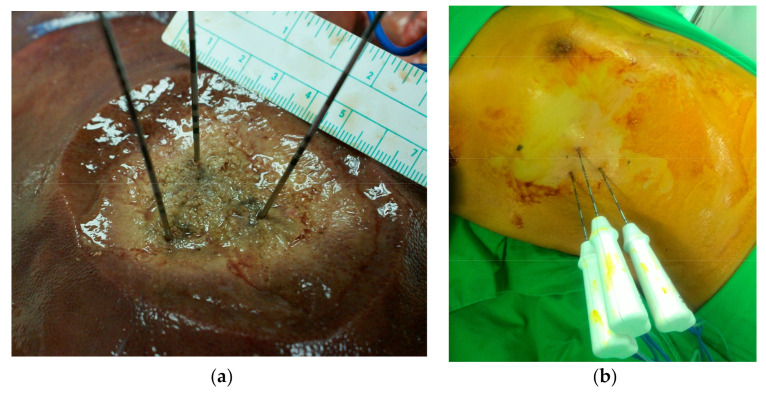
(**a**) multielectrode RFA can enlarge the ablation zone to 5 cm in diameter; (**b**) percutaneous multielectrode RFA.

The RF generators used for local ablation were monopolar systems, including the Cool-tip RF system (Medtronic, Mansfield, MA, USA) and the Viva RF System (STARmed, Korea), both of which were equipped with a multielectrode RF switching controller. These systems allow the operation of up to three internally cooled electrodes during ablation. In all cases, RF electrodes with 3 cm exposed tips were individually placed within the targeted and/or index tumor. The goal was to place multiple electrodes at intervals of 2–2.5 cm in an intratumoral configuration to maximize coverage. Switching controllers provides independent control over delivery of the radiofrequency energy to each electrode, making it possible to create three energy sources using a single generator (Figure 2). Note that in switching monopolar mode, RF energy delivery is alternated among multiple electrodes. Following placement of a single electrode, ablation was performed until the impedance shut-off cycled at approximately 15 s. Each ablation session lasted 16–25 min, and the ablation zone measured 5–6 cm in diameter. Track ablation was performed during every repositioning and final removal of the RF electrodes to minimize bleeding and tumor seeding.

The primary endpoint in this study was overall survival (OS), which was defined as the period between the detection of liver metastases and the time of death or last follow-up whichever comes first. The secondary endpoints included recurrence-free survival (RFS), intrahepatic recurrence-free survival (IHRFS), complications following multi-RFA or hepatectomy treatment, the complete ablation rate, and the local recurrence rate. RFS was defined as the length of time from the diagnosis of liver metastasis to the first report of recurrence or death (whichever came first). IHRFS was defined as the length of time from the diagnosis of liver metastases to the first report of recurrence of intrahepatic metastases or death. We recorded only grade III–IV RFA-related complications in accordance with the Clavien–Dindo classification system. Complete treatment was defined as a complete lack of viable intrahepatic tumors in medical images within three months after the first multi-RFA or hepatectomy treatment for CRLM. Instances of local recurrence following multi-RFA or hepatectomy were recorded only if the liver metastases occurred within 2 cm of the previous treatment site.

Patient age, serum CEA levels, and tumor size were expressed as mean and standard deviations and compared using Student’s t-test or ANOVA, as appropriate. The numbers of tumors were expressed as median, minimum, and maximum, and were compared using the Mann–Whitney U-test. Variables were compared between the three groups using ANOVA. OS was calculated using the Kaplan–Meier method and compared using the log-rank test. Univariate analysis of OS was performed using Cox regression analysis.

Propensity scores matching (PSM) was applied to patients in the multi-RFA and hepatectomy groups using a logistic regression model that included the following five covariates: ECOG status, primary lymph node status, diameter of the largest metastasis, usage of neoadjuvant chemotherapy, and usage of anti-VEGF agents. A 1:1 matching ratio between the two groups was set using the nearest-neighbor method (caliper = 0.15).

All factors shown to be significant in univariate analysis were incorporated into multivariate analysis with backward stepwise selection process. A *p*-value <0.05 was considered statistically significant. All statistical analyses were performed using SPSS v. 26 (IBM Corp., Armonk, NY, USA)

## 3. Results

### 3.1. Clinicopathological Characteristics

The 132 patients included in the final study cohort were categorized into three groups according to the initial treatment for liver metastases: multi-RFA, hepatectomy, and systemic treatment only. Before the PSM was applied, significant differences were observed among the three groups in terms of presence of comorbidities, the presence of extrahepatic metastases at the time of CRLM, and the initial serum CEA levels (Table 1). Anti-VEGF agents were less frequently used in the multi-RFA group than in the other groups. There were no significant differences in the number of liver tumors between the three groups; however, significant differences in the total diameter of the liver tumors were observed (Table 2). The median duration of hospitalization was two days in the multi-RFA group and nine days in the hepatectomy group (*p* = 0.169). Complete ablation was achieved in 61.8% of patients in the multi-RFA group, and complete resection was achieved in 78.9% of patients in the resection group (*p* = 0.227); these rates did not differ significantly, indicating there was no observable difference in the treatment efficacy of multi-RFA and resection. 

Intrahepatic recurrence occurred in 54 (79.6%) cases in the multi-RFA group and 19 (57.6%) cases in the hepatectomy group. Note that the rate of intrahepatic recurrence was higher in the multi-RFA group (*p* = 0.021). Among the patients who developed intrahepatic recurrence, 36 (52.9%) developed local recurrence close to the previous ablation zone in the multi-RFA group, whereas 9 (26.5%) developed local recurrence in the hepatectomy group; this difference was significant (*p* = 0.011). As shown in Table 3, extrahepatic recurrence was common in both groups, occurring in 50 (73.5%) patients in the multi-RFA group and 26 (76.5%) patients in the hepatectomy group. The rates of grade III–IV complications according to the Clavien–Dindo classification system were 1.5% in the multi-RFA group and 5.9% in the resection group (*p* = 0.191). Most complications were intra-abdominal abscesses and were successfully managed by drainage.

**Figure 3:**
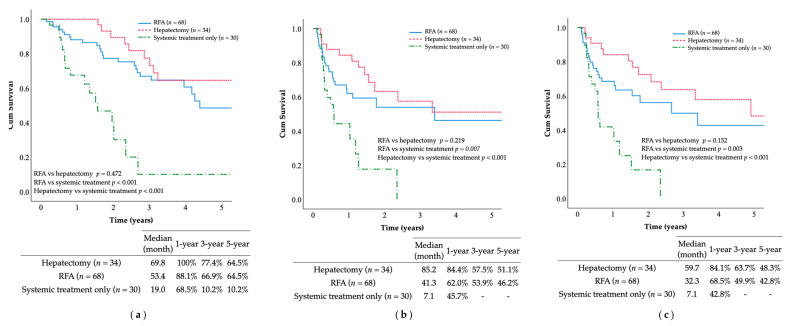
(**a**) The OS rates of the multi-RFA and hepatectomy groups were not significantly different and were superior to that of the systemic treatment only group (*p* < 0.001); (**b**) the RFS rates of the multi-RFA and hepatectomy groups were not significantly different and were superior to that of the systemic treatment only group (*p* = 0.007); (**c**) the IHRFS rates of the multi-RFA and hepatectomy groups were not significantly different and were superior to that of the systemic treatment only group (*p* = 0.003).

The 5-year OS rate and median OS duration after the median follow-up of 59.8 months were as follows: hepatectomy group (54.9%, 69.8 months), multi-RFA group (50.7%, 53.4 months), and systemic treatment only group (10.2%, 19.1 months). Patients receiving multi-RFA or hepatectomy were more likely to achieve long-term survival than those receiving systemic treatment only, as indicated by the significant differences in OS (*p* =< 0.001; Figure 3). Similar findings were observed in terms of RFS and IHRFS. The multi-RFA and hepatectomy groups also outperformed the systemic treatment only group, with the following median RFS and IHRFS durations: hepatectomy group (85.2, 59.7 months), multi-RFA group (41.3, 32.3 months), and systemic treatment only group (7.1, 7.1 months). The 5-year RFS and IHRFS rates were: hepatectomy group (51.1%, 48.3%), multi-RFA group (46.2%, 42.8%), and systemic treatment only group (0%, 0%). Patients receiving multi-RFA or hepatectomy achieved longer-term survival than patients who received systemic treatment only in terms of RFS (*p* < 0.001) and IHRFS (*p* < 0.001). However, no statistically significant differences were observed between the multi-RFA and hepatectomy groups in terms of OS (*p* = 0.472), RFS (*p* = 0.219), or IHRFS (*p* = 0.152).

### 3.2. Predictive Factors of Overall Survival in Patients with Multi-RFA vs. Hepatectomy

In our comparison of multi-RFA and hepatectomy, the ECOG status (*p* = 0.014), presence of comorbidities (*p* = 0.046), high tumor grade (*p* < 0.001), initial metastasis (*p* = 0.002), BRAF-mutated tumors (*p* = 0.015), and a history of RT (*p* = 0.002) were predictors of poorer OS. Multivariate analysis conducted using the aforementioned factors revealed that initial primary tumor M-positive status (*p* < 0.001), BRAF mutations (*p* = 0.003), and previous RT (*p* = 0.005) were significant predictors of a poorer prognosis in terms of OS (Table 4). Tumor grade was excluded because of missing data.

### 3.3. Predictive Factors of Overall Survival in Patients with Multi-RFA vs. Systemic Treatment Only

Univariate analysis of the multi-RFA and systemic treatment only groups revealed that the implementation of multi-RFA (*p* < 0.001), BRAF-mutated tumors (*p* = 0.004), multiple organ metastasis (*p* = 0.013), and a history of RT (*p* = 0.017) were predictors of poor prognosis for OS. Multivariate analysis revealed that only the implementation of multi-RFA (*p* < 0.001) and BRAF-mutated tumors (*p* < 0.001) were statistically significant predictors of a poor prognosis in terms of OS (Table 5). 

### 3.4. Overall Survival in Patients with and without Extrahepatic Metastasis

**Figure 4:**
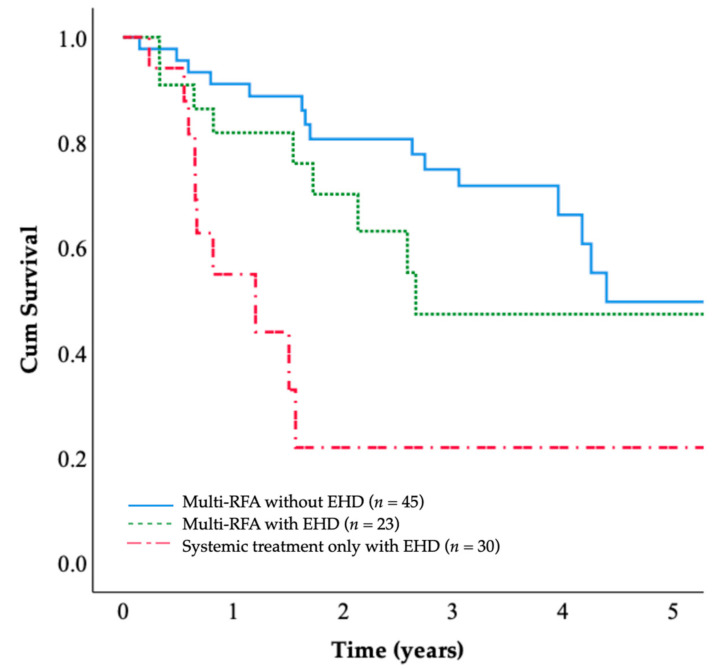
The OS rates of patients with or without extrahepatic metastasis were not significantly different (*p* = 0.134) and were superior to that of the systemic treatment only group (*p* < 0.001).

The patients in the multi-RFA group were classified according to whether they presented extrahepatic disease at the time of CRLM. The 5-year OS rates were 49.7% for the multi-RFA group without extrahepatic disease and 49.3% for the multi-RFA group with extrahepatic disease. The OS of patients with extrahepatic disease (*n* = 23) was comparable to that of patients without extrahepatic disease (*n* = 45), and the difference was not significant (*p* = 0.134; Figure 4); these OS rates were both higher than the OS rate of the systemic treatment only group.

### 3.5. Propensity Score Matching for Patients Who Underwent Multi-RFA or Hepatectomy

After matching according to the propensity score, 26 patients each from multi-RFA and hepatectomy groups were included. The clinicopathological and liver tumor characteristics showed no significant difference between the two groups (Table 6 and Table 7).

**Figure 5:**
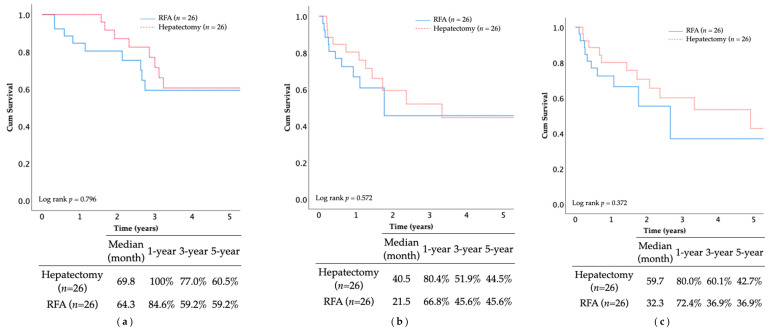
The OS rate (**a**), RFS rate (**b**), and IHRFS rate (**c**) of the multi-RFA and hepatectomy groups were still comparable after PSM.

The 5-year OS rate and median OS duration after the PSM were as follows: hepatectomy group (60.5%, 69.8 months), multi-RFA group (59.2%, 64.3 months) (*p* = 0.796; Figure 5). Similar findings were observed in RFS and IHRFS, suggesting comparable survival outcomes between the two groups without statistical significance.

The rates of complete treatment and major complications were not significantly different between the two groups (*p* = 0.734, *p* = 0.552, respectively).

Recurrence rate was comparable after PSM. Both intrahepatic and extrahepatic recurrence was similar between multi-RFA and hepatectomy groups (intrahepatic 76.9% vs. 53.8%, *p* = 0.080; extrahepatic 88.5% vs. 76.9%, *p* = 0.271). 

Univariate analysis of the multi-RFA and hepatectomy groups after PSM revealed that high tumor grade (*p* = 0.016) and primary tumor M status (*p* = 0.005) were predictors of poor prognosis for OS (Table 8). Multivariate analysis was conducted using the aforementioned factors and factors that showed significance before PSM; the analysis revealed that initial primary tumor M-positive status (*p* = 0.008) and high tumor grade (*p* = 0.007) were predictors of poor prognosis for OS.

## 4. Discussion

Metachronous liver metastases commonly develop among patients treated for colorectal cancer. In cases where radical liver intervention is feasible, the outcomes are generally satisfactory. RFA is increasingly being used as an alternative to liver metastasectomy because of the minimally invasive and repeatable nature of the procedure. Many studies have reported that surgical intervention is superior to RFA. Luo et al. retrospectively compared RFA and hepatectomy in patients with resectable colorectal liver oligometastases, and reported better OS in the resection group than the RFA group (53.6% vs. 42.5%); median intrahepatic recurrence-free survival (IHRFS) was also significantly longer in the resection group [19]. Wang et al. observed shorter disease-free survival in ablation groups (14 months) than in a resection group (22 months) [20]. However, improvements to ablative devices have enabled more complete and larger ablation zones to be achieved. Conventional single electrodes generate ablation zones limited to 3.0 cm, which precludes their use in larger liver tumors because of concerns of incomplete ablation or an insufficient margin. However, larger liver tumors can now be safely ablated using multiple electrodes with a switching controller [16], and systems featuring switching-controlled modes permit the delivery of higher radiofrequency currents through multiple electrodes, resulting in an ablation zone exceeding 5 cm in less than 30 min [21]. Furthermore, multiple electrode systems permit “no-touch ablation techniques,” which can potentially reduce the risk of track seeding [22]. However, there are few controlled studies of local ablation with new devices. The COLLISION trial (Colorectal Liver Metastases: Surgery vs. Thermal Ablation), which is comparing surgical resection with new thermal ablation in patients with resectable CRLM, is still ongoing [23]. The results of that trial may help to clarify the survival benefits of modern ablative devices in patients with CRLM.

The current study confirms that, for patients with resectable metachronous CRLM, the outcomes achieved by multielectrode RFA were comparable to those of hepatectomy in terms of OS, RFS, and IHRFS. Note that this study included tumors as large as 6 cm and cases with up to five liver metastases. Multivariate analysis showed that the number and size of the tumors were not related to a poor prognosis, suggesting that we could extend the limits on the number of liver tumors and tumor size when selecting patients with CRLM for multi-RFA [7,11,13,15].

The median hospitalization time of the patients in the multi-RFA treatment group was two days, and the median number of treatments was only two. The complication rates were also low after multi-RFA. These results confirm that multi-RFA is an effective, minimally invasive, well-tolerated treatment that can be repeated multiple times.

Despite leading to significantly higher intrahepatic recurrence rates, the OS and recurrence-free survival rates after multi-RFA were comparable to those of hepatectomy. In other words, minimally invasive multi-RFA treatment can be performed repeatably in patients with local recurrence, and the repeatability of multi-RFA might be the key to counterbalancing the high recurrence rates of CRLM.

When we evaluated the outcomes of patients with extrahepatic metastasis, the survival outcomes of this subgroup of the multi-RFA group significantly exceeded those of patients with extrahepatic metastasis in the systemic treatment only group. Thus, it may be necessary to reappraise the role of local interventions for CRLM in patients with extrahepatic metastasis, and aggressive liver-directed treatment should be encouraged.

We tried to balance the baseline difference between the multi-RFA and hepatectomy groups by introducing PSM, and the result still demonstrated comparable survival benefits between the two groups without statistical significance. Also, after PSM, no statistically significant difference was found in not only treatment completion and complication rates, but intra- and extrahepatic recurrence rates, proving the competent role of multi-RFA as an alternative modality for CRLM.

This study is subject to a number of limitations. First, this was a retrospective study conducted in a single medical center; therefore, the treatment preferences may be biased. Second, the sample size was relatively small, particularly when focusing on metachronous resectable CRLM and even smaller after PSM was implemented. Finally, the fact that a number of patients did not undergo molecular testing may bias the interpretation of our results.

In conclusion, this study demonstrated that multielectrode RFA can improve OS, RFS, and IHRFS in patients with metachronous CRLM and achieve comparable results to hepatectomy. Moreover, multielectrode RFA improved survival in metachronous CRLM, even in the presence of extrahepatic metastasis.

## Figures and Tables

**Table 1:** Clinicopathological characteristics before PSM.

Characteristics	Multi-RFA (*n* = 68)	Hepatectomy (*n* = 34)	Systemic Treatment Only (*n* = 30)	*p*-Value
Age (years) *	61.65 ± 11.99	61.03 ± 10.60	62.20 ± 14.79	0.931
Gender (male)	37 (54.4)	21 (61.8)	16 (53.3)	0.740
ECOG status 0	62 (91.2)	31 (91.2)	28 (93.3)	0.933
Comorbidities	38 (55.9)	13 (38.2)	22 (73.3)	0.018 ^‡^
Tumor location				0.464
Cecum	2 (2.9)	0	0	
Ascending	11 (16.2)	7 (21.2)	6 (20.7)	
Transverse	3 (4.4)	2 (6.1)	1 (3.4)	
Descending	3 (4.4)	2 (6.1)	4 (13.8)	
Sigmoid	16 (23.5)	10 (30.3)	8 (27.6)	
Rectosigmoid	6 (8.8)	4 (12.1)	5 (17.2)	
Rectum	27 (39.7)	8 (24.2)	5 (17.2)	
High tumor grade	2/54 (3.7)	0/19	2/23 (8.7)	0.369
T stage				0.059
1	2 (2.9)	2 (7.1)	0	
2	9 (13.2)	3 (10.7)	2 (6.7)	
3	51 (75.0)	20 (71.4)	21 (70.0)	
4	5 (7.4)	3 (10.7)	7 (23.3)	
N stage				0.522
0	19 (27.9)	12 (38.7)	9 (30.0)	
1	31 (45.6)	11 (35.5)	9 (30.0)	
2	18 (26.5)	8 (25.8)	12 (40.0)	
M stage				0.238
0	64 (94.1)	30 (90.9)	25 (83.3)	
1	4 (5.9)	3 (9.1)	5 (16.7)	
EGFR	8/10 (80.0)	12/12 (100)	8/11 (72.7)	0.178
KRAS	18/49 (36.7)	12/27 (44.4)	12/22 (59.1)	0.219
NRAS	2/16 (12.5)	0/6	1/2 (50)	0.198
BRAF	1/46 (2.2)	1/24 (4.2)	0/18	0.676
Unilateral CRLM	58 (85.3)	30 (88.2)	25 (83.3)	0.854
EHD at CRLM ^†^	23 (33.8)	12 (35.3)	18 (60.0)	0.041 ^‡^
CEA *	11.19 ± 19.25	6.23 ± 8.84	136.30 ± 264.64	0.004 ^‡^
CA19-9 *	80.50 ± 232.21	16.91 ± 16.52	63.22 ± 115.12	0.667
CEA at CRLM *^,†^	21.08 ± 37.31	15.37 ± 23.85	357.25 ± 1380.15	0.104
CA19-9 at CRLM *^,†^	350.46 ± 1014.60	22.64 ± 19.85	206.09 ± 278.05	0.545
Neoadjuvant Chemotherapy	10 (14.7)	1 (2.9)	3 (10.0)	0.193
Anti-VEGF agent	26 (38.2)	25 (78.1)	21 (70.0)	<0.001 ^‡^
Anti-EGFR agent	24 (35.8)	5 (16.1)	4 (13.3)	0.023 ^‡^

Values in parentheses are percentages, unless otherwise indicated. * Values are mean ± S.D. ^†^ EHD at CRLM is defined as the presence of extrahepatic metastasis at the time of diagnosis of liver metastasis. ^‡^ *p* < 0.05.

**Table 2:** Characteristics of initial liver tumor metastases.

Characteristics	Multi-RFA (*n* = 68)	Hepatectomy (*n* = 34)	Systemic Treatment Only (*n* = 30)	*p*-Value
Initial number	1 (1–6)	1 (1–6)	1 (1–4)	0.666
Largest tumor diameter	28.69 ± 13.82	36.13 ± 20.44	22.60 ± 13.16	0.003 *
Total diameter	37.32 ± 20.64	46.53 ± 29.25	31.30 ± 20.91	0.026 *
Initial number	1 (1–6)	1 (1–6)	1 (1–4)	0.666

Tumor number is expressed as median, minimum, and maximum. Tumor diameter is expressed as median ± S.D. (mm). * *p* < 0.05.

**Table 3:** Characteristics of recurrence after multi-RFA vs. hepatectomy.

Characteristics	Multi-RFA (*n* = 68)	Hepatectomy (*n* = 34)	*p*-Value
Intrahepatic recurrence	54 (79.4)	19 (57.6)	0.021 *
Local recurrence ^†^	36 (52.9)	9 (26.5)	0.011 *
Extrahepatic recurrence	50 (73.5)	26 (76.5)	0.748
Intrahepatic recurrence	54 (79.4)	19 (57.6)	0.021 *

* *p* < 0.05. ^†^ Local liver recurrence was defined as recurrence within 2 cm of the previous surgical area.

**Table 4:** Univariate and multiple variate analysis of multi-RFA vs. hepatectomy.

Variable	Univariate		Multiple Variate	
	Risk Ratio (95% CI)	*p*-Value	Risk Ratio (95% CI)	*p*-Value
Hepatectomy versus multi-RFA	1.284 (0.649–2.539)	0.473		
Age (≥65)	1.859 (0.972–3.558)	0.061		
Gender (male)	0.754 (0.397–1.432)	0.388		
ECOG (yes)	3.349 (1.271–8.820)	0.014 *	0.698 (0.087–5.594)	0.735
Comorbidity (yes)	1.955 (1.012–3.779)	0.046 *	1.327 (0.500–3.520)	0.570
Tumor location	1.707 (0.876–3.326)	0.116		
High tumor grade	39.844 (6.554–242.227)	<0.001 *	N/A	
T (T4)	0.882 (0.118–6.587)	0.902		
N (positive)	2.064 (0.998–4.271)	0.051		
M (positive)	4.753 (1.755–12.874)	0.002 *	14.45 (3.49–59.84)	<0.001 *
EGFR (mutant)	0.231 (0.024–2.259)	0.208		
KRAS (mutant)	1.003 (0.467–2.156)	0.993		
NRAS (mutant)	12.649 (0.776–206.139)	0.075		
BRAF (mutant)	20.50 (1.81–231.97)	0.015 *	272.2 (6.78–10,920)	0.003 *
Bilateral metastasis	1.373 (0.533–3.538)	0.512		
EHD at CRLM	1.699 (0.882–3.273)	0.113		
Neoadjuvant chemotherapy	0.963 (0.294–3.153)	0.951		
Anti-VEGF (yes)	0.892 (0.470–1.693)	0.726		
Anti-EGFR (yes)	0.711 (0.324–1.563)	0.396		
TACE/Y90 (yes)	0.882 (0.310–2.508)	0.814		
RT (yes)	2.871 (1.475–5.589)	0.002 *	4.834 (1.610–14.512)	0.005 *
Initial no (≥4)	0.047 (0.000–1194.315)	0.556		
Largest diameter (≥30 mm)	1.274 (0.669–2.425)	0.461		
Total diameter (≥50 mm)	1.441 (0.343–6.056)	0.618		
CEA (≥30 ng/mL)	1.393 (0.183–10.582)	0.749		
CA19-9 (≥100 U/mL)	2.118 (0.463–9.688)	0.333		
CEA at CRLM (≥30 ng/mL)	1.311 (0.528–3.254)	0.559		
CA19-9 at CRLM (≥100 U/mL)	2.390 (0.847–6.746)	0.100		
Time to CRLM (> 730 days)	0.949 (0.481–1.875)	0.880		

* *p* < 0.05.

**Table 5:** Univariate and multiple variate analysis of multi-RFA vs. systemic treatment only.

Variable	Univariate		Multiple Variate	
	Risk Ratio (95% CI)	*p*-Value	Risk Ratio (95% CI)	*p*-Value
Multi-RFA use (yes)	0.267 (0.140–0.510)	<0.001 *	0.111 (0.036–0.336)	<0.001 *
Age (≥65)	1.034 (0.560–1.910)	0.914		
Gender (male)	0.961 (0.526–1.754)	0.896		
ECOG (yes)	1.902 (0.672–5.382)	0.226		
Comorbidity (yes)	1.879 (0.977–3.611)	0.059		
Tumor location	1.307 (0.701–2.438)	0.400		
High tumor grade	2.614 (0.613–11.138)	0.194		
T (T4)	1.205 (0.366–3.963)	0.759		
N (positive)	1.845 (0.907–3.755)	0.091		
M (positive)	1.212 (0.367–4.006)	0.752		
EGFR (mutant)	0.760 (0.146–3.961)	0.744		
KRAS (mutant)	0.766 (0.347–1.689)	0.509		
NRAS (mutant)	7.746 (0.453–132.374)	0.157		
BRAF (mutant)	60.49 (3.78–967.23)	0.004 *	336.53 (17.2–6579.5)	<0.001 *
Bilateral metastasis	1.087 (0.483–2.447)	0.840		
EHD at CRLM	2.152 (1.175–3.941)	0.013 *	1.902 (0.750–4.764)	0.170
Neoadjuvant chemotherapy	1.027 (0.431–2.446)	0.952		
Anti-VEGF (yes)	1.449 (0.794–2.643)	0.227		
Anti-EGFR (yes)	0.645 (0.315–1.320)	0.230		
TACE/Y90 (yes)	0.782 (0.278–2.198)	0.641		
RT (yes)	2.109 (1.141–3.900)	0.017 *	1.356 (0.554–3.323)	0.505
Initial no (≥4)	0.791 (0.106–5.873)	0.818		
Largest diameter (≥30 mm)	1.118 (0.608–2.054)	0.720		
Total diameter (≥50 mm)	0.873 (0.447–1.707)	0.692		
CEA (≥30 ng/mL)	1.542 (0.329–7.223)	0.583		
CA19-9 (≥100 U/mL)	2.299 (0.632–8.365)	0.207		
CEA at CRLM (≥30 ng/mL)	1.566 (0.696–3.527)	0.279		
CA19-9 at CRLM (≥100 U/mL)	2.145 (0.801–5.747)	0.129		
Time to CRLM (> 730 days)	1.715 (0.913–3.221)	0.094		

* *p* < 0.05.

**Table 6:** Clinicopathological characteristics after PSM.

Characteristics	Multi-RFA (*n* = 26)	Hepatectomy (*n* = 26)	*p*-Value
Age (years) *	63.5 ± 12.7	62.6 ± 10.3	0.793
Gender (male)	15 (57.7)	14 (53.8)	0.780
ECOG status 0	21 (80.8)	23 (88.5)	0.442
Comorbidities	15 (57.7)	10 (38.5)	0.165
Tumor location			0.621
Cecum	2 (7.7)	0	
Ascending	5 (19.2)	7 (26.9)	
Transverse	2 (7.7)	2 (7.7)	
Descending	1 (3.8)	1 (3.8)	
Sigmoid	6 (23.1)	9 (34.6)	
Rectosigmoid	1 (3.8)	2 (7.7)	
Rectum	9 (34.6)	5 (19.2)	
High tumor grade	1 (4.5)	0	0.360
T stage			0.574
1	0	1 (4.3)	
2	5 (19.2)	3 (13.0)	
3	17 (65.4)	17 (73.9)	
4	4 (15.4)	2 (8.7)	
N stage			0.515
0	9 (34.6)	11 (42.3)	
1	13 (50.0)	9 (34.6)	
2	4 (15.4)	6 (23.1)	
M stage			0.638
0	24 (92.3)	23 (88.5)	
1	2 (7.7)	3 (11.5)	
EGFR	2/2	9/9	
KRAS	12/19 (63.2)	10/21 (47.6)	0.324
NRAS	1/5 (20.0)	0/5	0.292
BRAF	0	0	
Unilateral CRLM	21 (80.8)	23 (88.5)	0.442
EHD at CRLM	12 (46.2)	11 (42.3)	0.780
CEA *	9.95 ± 15.3	6.2 ± 8.8	0.453
CA19-9 *	175.3 ± 380.7	16.9 ± 16.5	0.205
CEA at CRLM *	17.7 ± 24.2	15.6 ± 27.3	0.813
CA19-9 at CRLM *	183.7 ± 341.8	22.8 ± 21.2	0.204
NeoadjuvantChemotherapy	2 (7.7)	1 (3.8)	0.552
Anti-VEGF agent	20 (76.9)	19 (73.1)	0.749
Anti-EGFR agent	8 (32.0)	5 (20.0)	0.333

Values in parentheses are percentages, unless otherwise indicated. * *p* < 0.05.

**Table 7:** Characteristics of initial liver tumor metastases after PSM.

Characteristics	Multi-RFA (*n* = 28)	Hepatectomy (*n* = 28)	*p*-Value
Initial number	1 (1–4)	1 (1–4)	0.369
Largest tumor diameter	32.7 ± 14.9	32.8 ± 18.8	0.971
Total diameter	42.4 ± 20.8	41.5 ± 27.0	0.901

Tumor number is expressed as median, minimum, and maximum. Tumor diameter is expressed as median ± S.D. (mm).

**Table 8:** Univariate analysis of multi-RFA vs. hepatectomy after PSM.

Variable	Univariate		Multiple Variate	
	Risk Ratio (95% CI)	*p*-Value	Risk Ratio (95% CI)	*p*-Value
Hepatectomy versus multi-RFA	1.118 (0.479–2.608)	0.796		
Age (≥65)	1.416 (0.587–3.416)	0.439		
Gender (male)	0.905 (0.389–2.106)	0.817		
ECOG (yes)	2.738 (0.885–8.476)	0.081		
Comorbidity (yes)	1.158 (0.487–2.756)	0.740		
Tumor location	2.092 (0.830–5.276)	0.118		
High tumor grade	18.994 (1.722–209.497)	0.016 *	29.733 (2.535–348.749)	0.007 *
T (T4)	1.572 (0.191–12.959)	0.674		
N (positive)	1.590 (0.662–3.818)	0.300		
M (positive)	5.416 (1.682–17.443)	0.005 *	5.295 (1.547–18.120)	0.008 *
EGFR (mutant)	N/A	N/A		
KRAS (mutant)	0.978 (0.376–2.545)	0.964		
NRAS (mutant)	N/A	N/A		
BRAF (mutant)	N/A	N/A		
Bilateral metastasis	0.842 (0.194–3.644)	0.818		
EHD at CRLM	1.528 (0.661–3.531)	0.321		
Neoadjuvant chemotherapy	1.179 (0.156–8.910)	0.873		
Anti-VEGF (yes)	0.892 (0.327–2.437)	0.824		
Anti-EGFR (yes)	0.696 (0.232–2.088)	0.518		
TACE/Y90 (yes)	0.829 (0.243–2.826)	0.764		
RT (yes)	0.047 (0.000–3538.623)	0.593		
Initial no (≥4)	1.005 (0.432–2.337)	0.991		
Largest diameter (≥30 mm)	0.664 (0.242–1.823)	0.427		
Total diameter (≥50 mm)	1.733 (0.215–13.941)	0.605		
CEA (≥30 ng/mL)	1.968 (0.219–17.723)	0.546		
CA19-9 (≥100 U/mL)	0.247 (0.032–1.891)	0.178		
CEA at CRLM (≥30 ng/mL)	1.486 (0.310–7.114)	0.620		
CA19-9 at CRLM (≥100 U/mL)	0.739 (0.310–1.760)	0.495		
Time to CRLM (> 730 days)	1.118 (0.479–2.608)	0.796		

* *p* < 0.05.

## Data Availability

The data that support the findings of this study are available on request from the corresponding author.
